# Engineered measles virus Edmonston strain used as a novel oncolytic viral system against human hepatoblastoma

**DOI:** 10.1186/1471-2407-12-427

**Published:** 2012-09-25

**Authors:** Shu-Cheng Zhang, Wei-Lin Wang, Wei-Song Cai, Kai-Lei Jiang, Zheng-Wei Yuan

**Affiliations:** 1 Department of Pediatric Surgery, Major Laboratory of Chinese Health Ministry for Congenital Malformations, Shengjing Hospital of China Medical University, 36 Sanhao Street Heping District, Shenyang 110004, P.R. China; 2 Department of Medical Oncology, Shengjing Hospital of China Medical University, 36 Sanhao Street Heping District, Shenyang 110004, P.R. China

**Keywords:** Hepatoblastoma, Oncolytic, Measles virus Edmonston strain, CD46

## Abstract

**Background:**

Hepatoblastoma (HB) is the most common primary, malignant pediatric liver tumor in children. The treatment results for affected children have markedly improved in recent decades. However, the prognosis for high-risk patients who have extrahepatic extensions, invasion of the large hepatic veins, distant metastases and very high alpha-fetoprotein (AFP) serum levels remains poor. There is an urgent need for the development of novel therapeutic approaches.

**Methods:**

An attenuated strain of measles virus, derived from the Edmonston vaccine lineage, was genetically engineered to produce carcinoembryonic antigen (CEA). We investigated the antitumor potential of this novel viral agent against human HB both in vitro and in vivo.

**Results:**

Infection of the Hep2G and HUH6 HB cell lines, at multiplicities of infection (MOIs) ranging from 0.01 to 1, resulted in a significant cytopathic effect consisting of extensive syncytia formation and massive cell death at 72–96 h after infection. Both of the HB lines overexpressed the measles virus receptor CD46 and supported robust viral replication, which correlated with CEA production. The efficacy of this approach in vivo was examined in murine Hep2G xenograft models. Flow cytometry assays indicated an apoptotic mechanism of cell death. Intratumoral administration of MV-CEA resulted in statistically significant delay of tumor growth and prolongation of survival.

**Conclusions:**

The engineered measles virus Edmonston strain MV-CEA has potent therapeutic efficacy against HB cell lines and xenografts. Trackable measles virus derivatives merit further exploration in HB treatment.

## Background

Hepatoblastoma (HB) is the most common primary, malignant liver tumor in children with an incidence of 0.7–1 case per million children [1-4]. The median age at diagnosis is 18 months; only 5% of the tumors are diagnosed after 4 years of age [5].

Although treatment strategies against HB have been established, they are constantly evaluated and revised through cooperative study groups worldwide [6-8]. The treatment results for affected children have markedly improved over the last decades due to the evolution of therapy from consisting of surgery alone to involving a multimodal approach that combines adjuvant chemotherapy regimens and surgery. Recent international clinical studies have focused on the risk-adapted treatment of standard-risk patients with potentially resectable tumors and high-risk patients with unresectable tumors associated with extrahepatic extensions, invasion of the large hepatic veins, distant metastases and very high alpha-fetoprotein (AFP) serum-levels [2-4]. To date, about 92% of the patients in the standard-risk group can be cured by combining neo-adjuvant chemotherapy and surgery, whereas approximately 60% of all high-risk patients survive [2-4]. One problem that is especially present in the latter group is multidrug resistance after a number of chemotherapy courses [1,5]. Thus, the implementation of new, efficient drugs into future therapeutic regimens is especially critical.

Oncolytic therapy, which uses replication-competent viruses that replicate in the tumor cells and kill the cells lytically has limited side effects. This therapy has shown great potential in the treatment of multiple tumors such as lymphoma, ovarian cancer, mesothelioma, breast cancer, renal and hepatocellular carcinoma [9-15]. A large variety of oncolytic viruses have been engineered [16-22]. Among the many oncolytic virus systems, the attenuated Edmonston vaccine strain of the measles virus (MV-Edm) has proven safe and effective [23-27]. It exerts its cytopathic effect (CPE) by fusing infected cells with the surrounding cells, forming multinucleated syncytia, which is followed by cell death by apoptotic or nonapoptotic mechanisms [24,26,28]. MV is a negative-strand RNA paramyxovirus, and it has been shown that MV-Edm preferentially fuses and kills cells overexpressing the CD46 receptor [29]. CD46 is a membrane-associated complement regulatory protein that is ubiquitously expressed on nucleated human cells [30,31]. Tumor cells frequently overexpress CD46 [32]. These mechanisms contribute to the tumor selectivity of MV-Edm.

In contrast to the wild-type virus, which can cause a potentially serious disease, the vaccine and the Edmonston strains of the measles virus have an excellent safety record, with millions of administered vaccine doses that have significantly decreased the incidence, morbidity and mortality of measles worldwide [33]. Another advantage of using the MV-Edm as a vector is that the virus may be effectively engineered to express soluble marker peptides, such as CEA and beta-HCG, which may be employed as real-time correlates of viral gene expression in vivo. Furthermore, this virus expresses membrane proteins, such as the sodium iodine symporter, which allows for radionuclide imaging-based assessment of the viral localization and spread over time [33,34]. The serum CEA level has been used as an effective surrogate of the viral gene expression, which correlates with viral growth both in vivo and in vitro [33]. The detection of CEA in serum is widely available through clinical assays. Therefore, measuring the serum CEA is a cost-effective method for monitoring viral gene expression after MV-CEA treatment. Furthermore, this method allows for repeated measurements with minimal risk to the patient.

In the current study, we evaluated the efficacy of recombinant MV-Edm that expresses the soluble extracellular N-terminal domain of human CEA (MV-CEA) against human HB cell lines and xenografts.

## Methods

### Cell culture

The human HB cell line Hep2G was obtained from the American Type Culture Collection (ATCC; Manassas, VA) and maintained in modified Eagle's medium (MEM) supplemented with 10% heat-inactivated fetal bovine serum (FBS) and 1% sodium pyruvate. The HUH6 cell line was kindly denoted by Professor Cai from the China Medical University. The normal human liver cell line L-02 was obtained from ATCC and maintained in MEM supplemented with 10% heat-inactivated fetal bovine serum (Sigma, Steinheim, Germany). The Vero African green monkey kidney cells (ATCC, CCL-81) used for the production of MV were maintained in DMEM supplemented with 5% FBS. All media used in this study contained 100 U/ml penicillin-streptomycin. Growth media, sera, and supplements were obtained from Gibco BRL (Grand Island, NY). All cells used in this study were cultured in a humidified atmosphere of 5% CO_2_ at 37°C.

### Viruses and infection assays

The construction of MV-CEA was carried out in our laboratory as described previously [33]. The reverse genetics system, described by Radecke was employed [35]. In summary, the NSe strain c-DNA infectious clone (derived from the MV-Edm vaccine lineage Seed B) [36] was engineered by inserting the human CEA gene upstream of the MV *N* gene. The titers of viral stocks were determined by 50% endpoint dilution assays (TCID 50) on Vero cells. For virus infection assays, 2 × 10^5^ cells were incubated with recombinant MV-Edm was diluted in 1.0 ml of Opti-MEM (Life Technologies, Inc. Shanghai, China) for 2 hours at 37°C. At the end of the incubation period, the virus was removed, and the cells were maintained in standard medium.

### Evaluation of CPEs in vitro

The Hep2G, HUH6 and L-02 cell lines were cultured in 24-well plates at a density of 2 × 10^5^ cells/well. The cells were infected with MV-CEA at a multiplicity of infection (MOI) of 1 or 0.1 in 0.2 ml of Opti-MEMI (GIBCO, Invitrogen, Shanghai, China) for 2 hours. The virus suspension was removed, and 1 ml of fresh medium was added to each well. At 96 hours after infection, the cells were gently washed twice with phosphate buffered saline (PBS), and the remaining cells were fixed with 0.5% glutaraldehyde in PBS for 15 minutes. Then, the cells were washed with PBS and stained with 0.1% crystal violet solubilized in 2% ethanol–distilled water. The stained product was subsequently washed twice with distilled water, air-dried, and then photographed.

### Cell proliferation assay

The Cell-Titer 96 Aqueous Non-Radioactive Cell Proliferation Assay (Promega, Madison, WI) was used in this study. Hep2G, HUH6 and L-02 cell lines were plated in 96-well plates at a density of 1 × 10^5^ cells/well. Twelve hours after seeding, the cells were infected with MV-CEA at an MOI of 0.1 for different time intervals and then incubated with 20 μl of the methanethiosulfonate (MTS) reagent for 2 hours at 37°C. The absorbance at 490 nm was recorded using an enzyme-linked immunosorbent assay (ELISA) plate reader.

### Assessment of MV replication in human HB cells

Cells from the human Hep2G, HUH6 and L-02 cell lines were seeded in 6-well plates at a density of 2.0 × 10^5^ cells/well. Twelve hours after plating, the cells were infected with each MV at an MOI of 0.1 in Opti-MEM I. The cells and supernatants were collected at different time intervals. The viruses were released by two cycles of freezing and thawing. The viral titers in the cells and supernatants were determined by CEA detection using a CEA ELISA kit (PBL Biomedical Laboratories) as per the manufacturer’s instructions.

### CEA analysis

For the in vivo experiments, blood samples were collected from mice by retro-orbital bleeding, and the serum was analyzed to determine the CEA concentration. For the in vitro experiments, the supernatant from the MV-CEA-infected and uninfected HB cells was collected and analyzed to determine the CEA concentration. The ELISA specific for CEA was performed using an ELISA kit (PBL Biomedical Laboratories) as per the manufacturer’s instructions.

### In vivo experiments

All procedures involving animals were approved by and performed according to guidelines of the Institutional Animal Care and Use Committee of the China Medical University. A 27-gauge needle was used to subcutaneously inject nude mice (purchased from the laboratory animal center of the China Medical University) with 5 × 10^6^ Hep2G cells/100 μL PBS in the right flank. Mice were examined daily for tumor growth. Tumor length, width, and height were measured with calipers. Tumor volume was calculated according to the formula width × width × height/2. When tumors reached a maximum diameter of 0.5 cm, the MV-CEA and UV-inactivated MV-CEA treatments were initiated by intratumoral injection (*n* = 8 each group). Animals were euthanized when the tumor diameter reached 1 cm or when 20% of the body weight was lost.

### Flow cytometry

The CD46 expression and the number of cells that died by apoptosis were determined by flow cytometry. To measure the CD46 expression, the cells were harvested with Cell Dissociation Buffer (GIBCO, Invitrogen), washed twice with PBS and incubated with a fluorescein isothiocyanate–labeled monoclonal mouse antihuman CD46, nectin 4 or isotype control antibodies (BD Biosciences, Pharmingen, US) for 1 hour on ice. The cells were washed twice with PBS. The cells were analyzed (10,000 cells per sample) using a FACScan (BD Biosciences, San Jose, CA). For the in vitro apoptosis assays, HB cells were plated in 6-well plates and treated with MV-CEA at an MOI of 0.1. Adherent and detached cells were harvested at 24, 48 and 72 hours after infection by centrifugation (1000 rpm) and were washed twice with cold PBS. The cell pellet was re-suspended in 1× binding buffer at a concentration of 1 × 10^6^ cells/ml. A total of 100 μl of the cell suspension was transferred into a flow cytometry tube. In the next step, 5 μl Annexin V-FITC and 10 μl PI were added into the cell suspension followed by gentle vortexing. The cells were incubated at room temperature for 15 minutes in the dark. An additional 400 μl of 1× binding buffer was added to each tube. Finally, the cells were analyzed using cell Quest software (BD Biosciences, San Jose, CA). All cells were made within the scope of the axes, and the gates were then set by drawing boundaries around the crowded subsets with 10^5^ cells that had an FSC-height >200 selected.

### Western blot analysis and ELISA

Infected cells were harvested and solubilized in a Nonidet P-40-based lysis buffer [20 mmol/L Tris (pH 7.4), 250 mmol/L NaCl, 1% Nonidet P-40, 1 mmol/L EDTA, 50 mg/ml leupeptin, and 1 mmol/L phenylmethylsulfonyl fluoride]. After incubating the cells on ice for 5 minutes, the cell lysates were clarified by centrifugation at 13,000 *g* for 30 minutes at 4°C. The protein concentrations in the lysates were quantified using the Multiskan spectrum (Thermo Scientific, Finland). The samples were separated on precast 4–12% gradient MOPS polyacrylamide gels (NOVEX, San Diego, CA) and then transferred to nitrocellulose membranes (BIO-RAD, Hercules, CA). The membranes were pretreated with Tris-buffered saline (TBS) containing 5% dry milk and 0.05% Triton X-100 (TBST) for 1 hour at room temperature and were then incubated with monoclonal antiproteolytic cleavage of the poly(ADPribose) polymerase (Biovision, Mountain View, CA) and rabbit anti-ß-actin (CHEMICON International, Temecula, CA) antibodies for 1 hour at room temperature. After several washes in TBST, the membranes were probed with rabbit or mouse peroxidase-conjugated secondary antibodies (Santa Cruz Biotechnology, Santa Cruz, CA) at room temperature for 1 hour. After a final wash with TBST, the immune-reactivity of the blots was detected using an enhanced chemiluminescence detection system (Amersham, Piscataway, NJ).

### Statistical methods

All the collected data were analyzed with SPSS13.0 software. The statistical analysis of the significance of the differences in survival between mice treated with recombinant MV-CEA and mice that did not receive MV-CEA was performed using the log-rank test in the JMP (John Macintosh Product) program. The analysis of flow cytometry apoptosis comparisons used t test.The significance level was set at *p* < 0.05.

## Results

### Overexpression of CD46 in human HB cells

**Figure 1:**
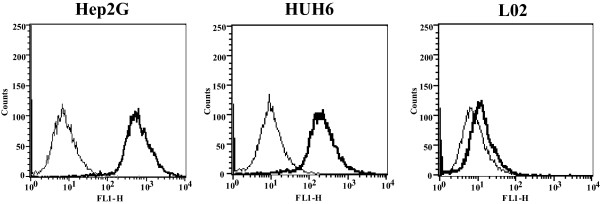
**Expression of the CD46 receptor in human HB cells and a normal liver cell line. **High levels of the CD46 receptor were observed in the human HB cell lines with the rate of 90.82% in Hep2G cells and 80.03% in HUH6 cells. A relatively low level of the CD46 receptor was detected in L-02 cells. Analyses were performed by flow cytometry using a monoclonal CD46 antibody.

The expression of CD46 in the human HB cell lines, Hep2G and HUH6, and in the human normal liver cell line L-02 was analyzed by flow cytometry with a FITC-labeled monoclonal antihuman CD46 antibody. Human HB cell lines express high levels of CD46 compared to the normal liver cell line L-02. The CD46 receptor was found in 90.82% of the Hep2G cells and 80.03% of the HUH6 cells. A relatively low level of 8.91% was demonstrated in L-02 (Figure 1).

### MV-CEA induces significant CPEs and exhibits an antitumor effect in human HB lines

**Figure 2:**
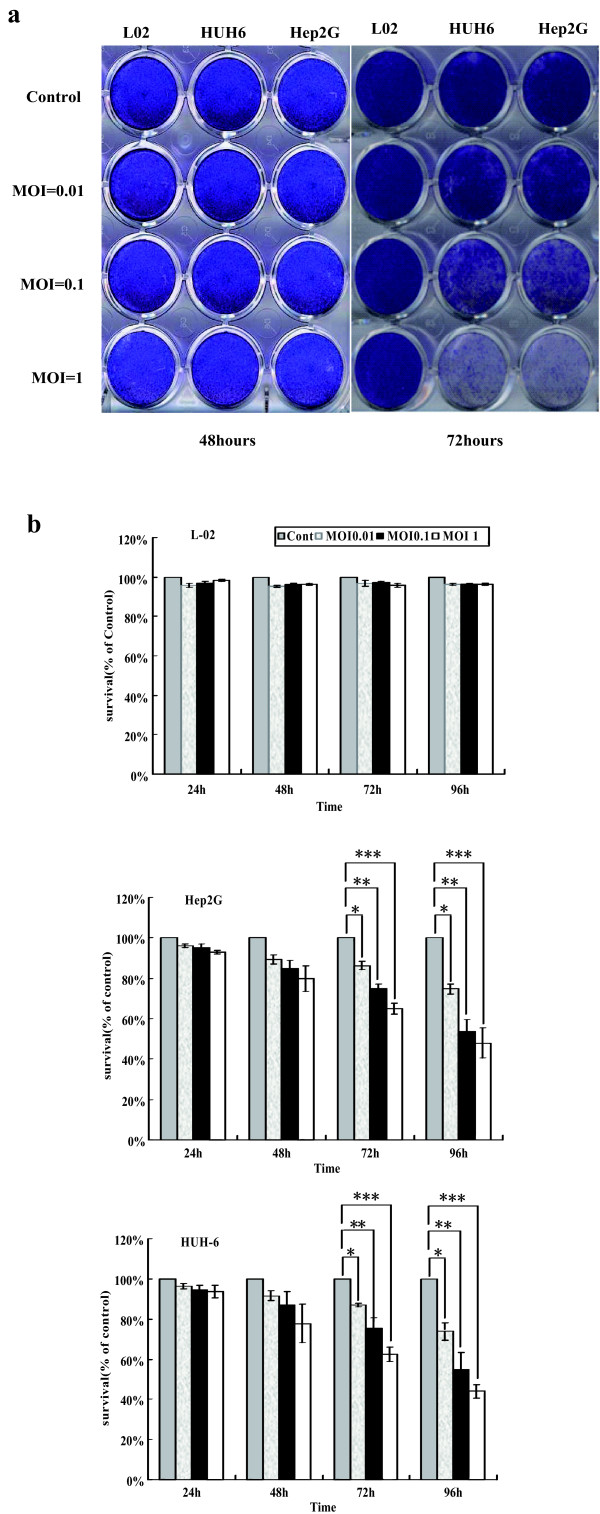
**Infectivity, induction of syncytia, and CPE of MV-Edm in human HB cells. **(**a**) Serial analysis to determine the CPE of recombinant MV-Edm was performed every 24 hours on the human HB cell lines Hep2G and HUH6 and normal liver cell line L-02. Seventy-two hours after infection at MOIs of 0.1 and 1, the cells were stained with crystal violet representing viable, attached cells. (**b**) The time course of cell viability of the human HB cell lines Hep2G and HUH6 and normal liver cell line L-02 (*n* = 8) after infection with recombinant MV-Edm at MOIs of 0.1 and 1 was analyzed using a Cell Titer 96 Aqueous nonradioactive cell proliferation assay kit. ***Group_MOI=1.0_*versus *Group _MOI=0_, P < 0.05; ** Group_MOI=0.1_*versus *Group _MOI=0_, P < 0.05. * Group_MOI=0.01_*versus *Group _MOI=0_, P < 0.05.

To determine the infectivity of the recombinant MV-CEA in human HB cells, the HB cell lines Hep2G and HUH6 and the normal liver cell line L-02 were infected with MV-CEA at MOIs of 1, 0.1 and 0.01 for 96 hours and then stained with crystal violet. Cell viability after MV-CEA infection was determined using the Cell-Titer 96 Aqueous Non-Radioactive Cell Proliferation Assay. Analyses were performed every 24 hours for 96 hours. MV-CEA demonstrated dramatic CPEs in an MOI-dependent manner. The CPEs appeared at 72 hours post infection with MV-CEA at an MOI of 0.1 in Hep2G and HUH6 cells. However, the normal liver cell line L-02 showed minimal CPEs after MV-CEA infection (Figure 2a).

Compared with the control, MV-CEA demonstrated a greater reduction in the proliferation of Hep2G and HUH6 cells from 72 to 96 hours at an MOI of 0.1. Reduction of viability was observed in the Hep2G and HUH6 cells within 72 hours after infection. Seventy-two hours after infection, the viability of the Hep2G cells was reduced to 74.67% at an MOI of 0.1 and to 86.33% at an MOI of 0.01. The HUH6 cells yielded similar results (Figure 2b).

### MV-CEA successfully replicates in human HB cell lines and induces cell lysis

**Figure 3:**
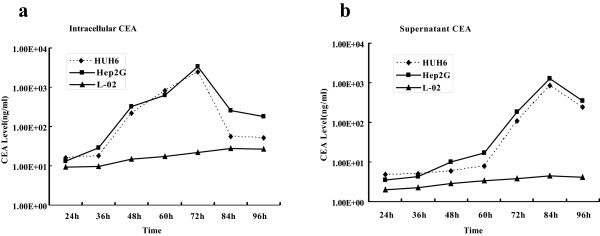
**Replication of MV-CEA in human HB cells. **CEA level of the (**a**) intracellular and (**b**) culture supernatant in the Hep2G cell line was detected by ELISA from 24 to 96 hours after infection at an MOI of 0.1. The intracellular CEA levels increased with time and peaked at 72 hours post infection, and the supernatant CEA level peaked at 84 hours post infection in Hep2G cells but not in L-02 cells. This phenomenon provides strong evidence for MV-CEA replication and cell lysis.

Hep2G, HUH6 and L-02 cells were plated on 6-well plates at a density of 2 × 10^5^ cells/well. The cells were infected with MV-CEA at an MOI of 0.1, and the supernatants and cells were collected from 24 to 96 hours postinfection. The intracellular viruses were released by two cycles of freezing/thawing. CEA levels revealed a time-dependent increase in MV mRNA in Hep2G and HUH6 cells, but not in the L-02 cell line. The intracellular CEA level peaked at 72 hours postinfection in the Hep2G and HUH6 cell lines (Figure 3a). In the culture supernatant, the CEA level peaked at 84 hours post infection (Figure 3b).

### MV-CEA infection induces significant apoptosis in human HB cell lines

**Figure 4:**
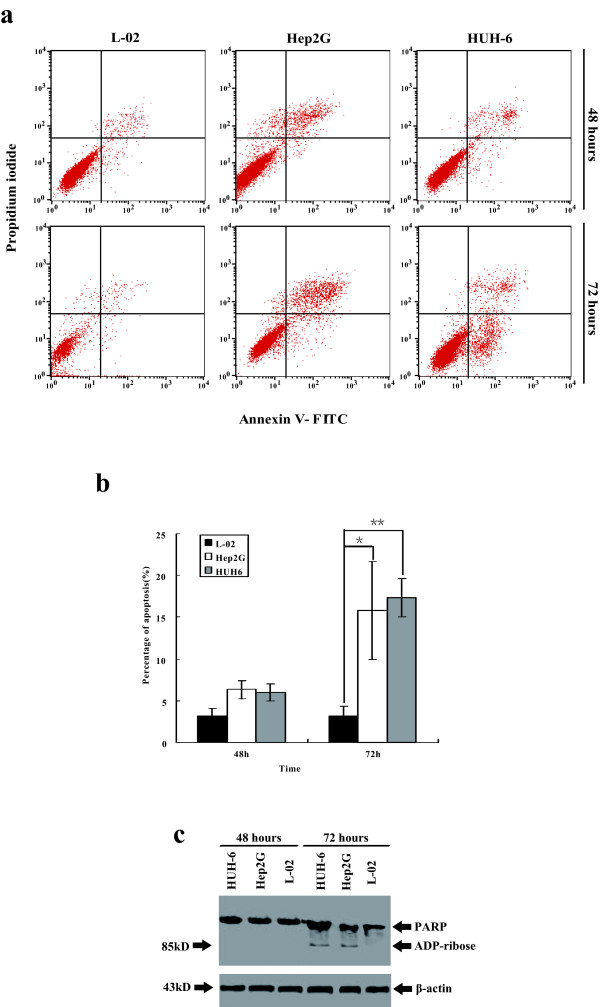
**Apoptosis induced by MV-CEA in the human Hep2G and HUH6 cells. **The cells were infected with MV-CEA at an MOI of 0.1. Adherent and detached cells were harvested at 24, 48 and 72 hours post infection. (**a**) The percentage of apoptotic cells was measured by FACS and is shown in this figure. In the L-02 cells, there was no dramatic apoptosis, but the Hep2G cells demonstrated dramatic apoptosis at 72 hours postinfection. (**b**)The significant difference is significant between the HB and L-02 groups but not between the two HB cell lines. * Group _Hep2G_*Versus *Group _L-02_, P < 0.01; **Group _HUH-6_*Versus *Group _L-02_, P < 0.01. (**c**) We further examined the poly(ADP-ribose) polymerase expression by Western blot and found that the 85-kd cleaved poly(ADP-ribose) polymerase fragment was expressed in the Hep2G and HUH-6 cells at 72 h after infection with MV-CEA. This finding was in agreement with the FACS results.

Hep2G, HUH6 and L-02 cells were infected with MV-CEA at an MOI of 0.1 and apoptotic cells, which also had Annexin V-FITC staining, were analyzed by propidium iodide staining and subsequent flow cytometry. Upon infection with MV-CEA, the number of apoptotic cells increased in a time-dependent manner. At an MOI of 0.1, MV-CEA induced apoptosis in 7.67% and 15.8% of the Hep2G cells and 7.3% and 17.35% of the HUH6 cells at 48 and 72 hours, respectively. However, at the same MOI, MV-CEA induced apoptosis in fewer than 5% of the L-02 cells. The difference is statistically significant between HB and L02 group (P < 0.05) but is not statistically significant between Hep2G and HUH6 cells (P > 0.05), indicating that MV-CEA induces significant apoptosis in human HB cells (Figure 4a,b). We further examined the poly(ADP-ribose) polymerase expression and found that the 85-kd cleaved poly(ADP-ribose) polymerase fragment was expressed in Hep2G cells at 48 h and 72 h after being infected with MV-CEA. This finding was in agreement with the FACS results (Figure 4c).

### Intratumoral administration of MV-CEA induces regression of HB xenografts and Can Be monitored by serial serum CEA concentrations

**Figure 5:**
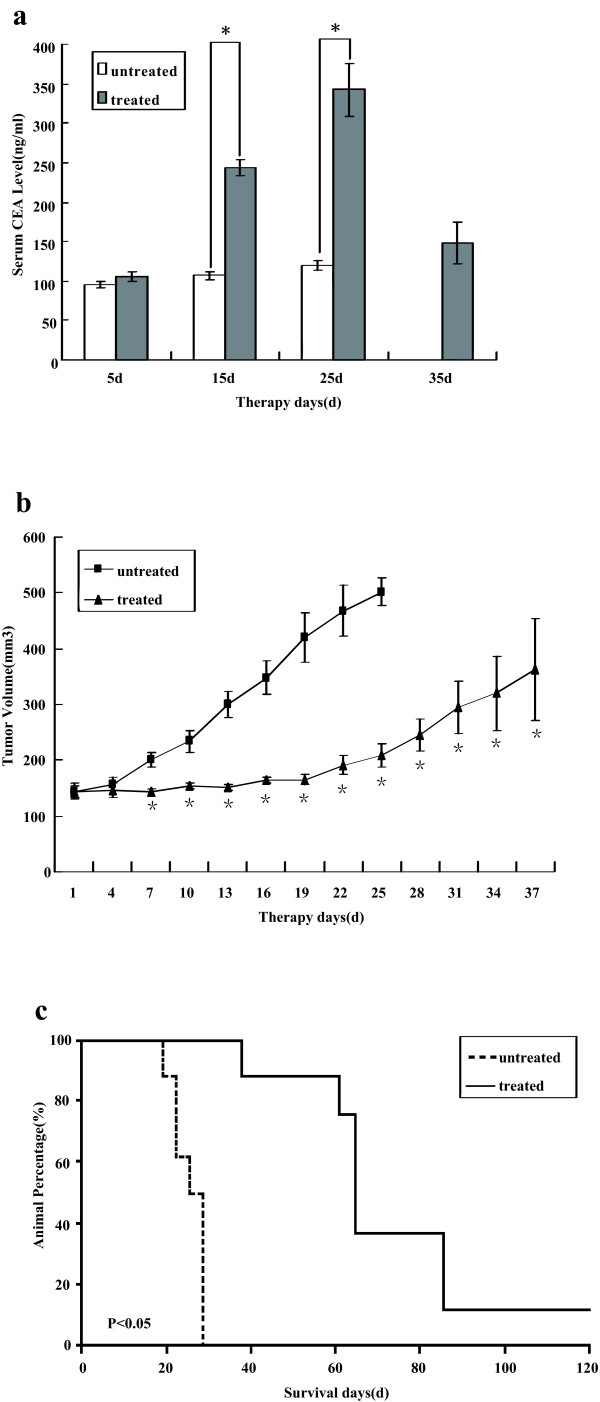
**The serum CEA concentrations and tumor suppression after intratumoral MV-CEA therapy of human HB xenografts. **Mice bearing HB xenografts (Hep2G) were injected intratumorally with 2 × 10^6^ TCID50 of MV-CEA every other day a total of five times (1.0 × 10^7^ total TCID50/mouse). Treatment groups (*n* = 8 per group) received active MV-CEA; the untreated group received UV-inactivated MV-CEA. (**a**) The time course of serum CEA concentration in HB xenograft-bearing mice after MV-CEA therapy. *Group _untreated_*versus *Group _treated_, P < 0.05; (**b**) The increase in tumor volume after initiation of the MV-CEA therapy. The data points are given as the median with positive standard error. *Group _untreated_*versus *Group _treated_, P < 0.05; (**c**) The Kaplan-Meier survival curves of the treated and untreated mice. The results show significant suppression of tumor growth in the MV-CEA treated animals (*p* < 0.05) and statistically significant prolongation of survival (*p* < 0.05).

To evaluate the potential use of recombinant MV-Edm for HB therapy, we first tested MV-CEA in a subcutaneous human HB xenograft model. Hep2G cells (2 × 10^6^ cells/mouse) were implanted in the right flanks of nude mice. When the maximum tumor diameter measured approximately 0.5 cm, each mouse was treated with a total of 5 doses of MV-CEA (1.0 × 10^7^ TCID50) or an equivalent dose of UV-inactivated MV-CEA for 10 days (defined as untreated, *n* = 8 per group). Serum CEA concentration, tumor volume, and survival curves are shown in Figure 5.

In the Hep2G xenograft models, the serum CEA concentrations could be detected as early as 4 days after initiating therapy; these concentrations increased over time. The CEA concentration reached its maximum on day 25 after the last viral dose. After reaching their maximum concentrations, the mean CEA levels started to decrease (Figure 5a). No CEA elevation was observed in the UV-inactivated MV-CEA-treated (defined as untreated) animals.

In the Hep2G cell line xenografts, the tumor-suppressive effect of MV-CEA first became apparent on day 7, and this therapeutic efficacy then increased over time, resulting in significant suppression of tumor growth and prolonged survival of treated animals (Figure 5b,c). The median survival of those treated with MV-CEA and of those treated with UV-inactivated MV-CEA was 72 and 25 days, respectively. The median survival of the MV-CEA-treated mice, with a 2.88-fold increase compared to the control group, was significantly longer than that of the control group (P < 0.05; Figure 5c). All mice in the control group had to be euthanized on day 28. In the MV-CEA-treated group, complete tumor regression was observed in one-eighth of treated mice (P < 0.05; Figure 5c).

## Discussion

Oncolytic therapy has reportedly excellent efficacy in the treatment of many tumors, such as lymphoma, ovarian cancer, mesothelioma, breast cancer and hepatocellular carcinoma [9-15]. Specifically, there are many advantages in using oncolytic therapy to treat liver malignancies; at some medical centers, oncolytic therapy has even been evaluated in clinical trials [37-39]. As a special subtype of hepatocellular carcinoma, HB originates from the liver embryonic tissues and has the potential for diverse differentiation. Many components, such as the epithelium, bone and cartilage, can be included within the tumor; as a result, HB differs from general hepatocellular carcinoma in its histological and the biological characteristics. Histologically, the tumors are divided into epithelial and mixed epithelial/mesenchymal subtypes. Tumor cells may appear with a wide variety of characteristics ranging from almost liver-cell-like to undifferentiated blastomal cells. The majority of HB cells are epithelial, consisting of embryonal and fetal cells. About 5% of the tumors belong to the small-cell undifferentiated subtype, which is associated with a worse prognosis [5,40]. Despite many advances in the use of oncolytic therapies for other liver malignancies, little is known about the use of oncolytic therapy in human HB. In this study, we report the potent therapeutic efficacy of oncolytic virus against human HB cells.

Currently, a large variety of oncolytic viruses are under evaluation in clinical trials [16-22]; the most common viruses tested are derived from the attenuated Edmonston vaccine strain of the measles virus [9-15], adenovirus [16,17], herpes simplex virus (HSV) [18,19], Newcastle disease virus (NDV) [20], parvovirus [21], and poliovirus [22]. The present results demonstrate that virotherapeutics can be used safely and efficiently as cancer therapies. For safety reasons, vaccine virus-derived virotherapeutics are of special interest (e.g., measles vaccine virotherapeutics) because they are approved for human use, have been applied millions of times with a longstanding excellent safety record, and exhibit a potent natural oncolytic activity [41,42]. As one of the most tested vaccines, the attenuated Edmonston vaccine strain of the measles virus has been well reported in hepatocellular carcinoma but not in HB treatment. MV is a replicating virus; therefore, the MV-Edm vaccine strain derivatives of MV can offer the potential advantage of increased dissemination in the tumor and of potentially enhanced therapeutic benefit compared to non-replicating viral or non-viral vector systems.

The MV enters the cells through the interaction of the H-glycoprotein with the MV receptors, CD150 (signaling lymphocyte-activation molecule, SLAM) and CD46 [41-43]. Of note, the wild-type measles virus enters more efficiently through the SLAM receptor, whereas the Edmonston vaccine strain of measles virus enters the cells predominantly through the CD46 receptor. The MV receptor CD46 (membrane cofactor protein) belongs to the family of membrane-associated complement regulatory proteins that serve as an important mechanism of self-protection against complement-mediated lysis. Tumor cells frequently overexpress CD46. These mechanisms contribute to the tumor selectivity of MV-Edm. The effectiveness of MV-Edm-mediated oncolysis is highly dependent upon the expression of the cellular attachment receptor CD46, which is expressed more frequently in human cancer cells than in normal cells. In this study, we have demonstrated that the measles virus vaccine strain derivative MV-CEA, which has been genetically engineered to produce CEA, has significant antitumor activity against HB as indicated by the CPE of MV-CEA on HB cell lines in vitro and the efficacy of MV-CEA in an HB xenograft model. Our data show that CD46 is overexpressed in HB cell lines compared to normal liver cells, and MV-CEA successfully infected human HB cells, resulting in transgene expression, syncytium formation, and tumor cell killing; therefore, we conclude that HB fulfills the requirements for viral uptake and selective cell fusion and killing.

It is likely that additional factors contribute to the MV-Edm tumor selectivity. In 2011, two independent groups reported the identification of a novel MV-Edm receptor, nectin 4 [44-46]. It is a tumor cell marker found in breast, lung and ovarian carcinomas and rendered cells susceptible to the MV-Edm. The transient knockdown of nectin 4 with siRNA abolished the wild-type MV infection in these cell lines. Similar results were also confirmed in the MV-Edm. The binding of the V domain of nectin 4 to MV-H has been considered a potential mechanism for the MV pathogenicity [44]. Also, a few studies have indicated that MV infection can occur via CD147 and virion-associated CypB, independent of MV-H [47]. Watanabe et al [47]. identified CypB as a binding partner of MV-N and further showed that the wild-type MV recognizes CD147 as a receptor on epithelial cells via the CypB that is incorporated into the virus particles. It is still not clear whether other MV strains, such as the MV-Edm, share a similar pathway. Although a variety of other receptors including H protein dependent or independent ones can be used for MV-Edm infection, it has been reported that their efficiency is far lower than CD46 [46]. The CD46 receptor is still the main player for MV-Edm spreading between cells. Therefore, it is not necessary to detect all of the other receptors one by one when a high level of CD46 is sufficient for demonstrating the oncolytic mechanism.

MV-CEA resulted in a strong CPE in vitro and in vivo. In this study, two human HB cell lines, Hep2G and HUH6, were used. These two lines have features that are the most characteristic of human HB and have been widely used in HB-related investigations [40,48]. Although the HB subtype and the risk level (either standard-risk or high-risk) induced by these two lines are still not verified, it is not critical for the oncolytic virus to be used in the HB biotherapy. Only cells that express high levels of the CD46 receptor are infected by MV-Edm and lytically killed. This statement was verified in the present study; both of the tested cell lines were susceptible to the cytotoxic effect of MV-CEA but differed in their cell death kinetics. The Hep2G cells were eliminated very efficiently and quickly, whereas the cytotoxic effect of MV-CEA on the HUH6 cells was observed later. The HB cell lines used in our study showed variable susceptibility to the cytotoxic effect of MV-CEA, most likely resembling the situation in primary human tumors, which are composed of heterogeneous tumor cell populations [5]. Both cell lines express comparable levels of CD46 but differ in other aspects, such as their histological subtypes. Differences in the components of these two lines or other not-yet-identified factors in the process of measles-induced cytotoxicity could explain the differences in susceptibility.

As one of the most common causes of cell death, apoptosis has been well described in other malignancies. It has been reported that MV-Edm can induce apoptosis in both the tumor cells and the syncytia by a series of signal pathways, such as the Fas-associated death domain (FADD), protein kinase C (PKC), and the janus kinase-signal transducer and activator of transcription (JAK-STAT) signaling pathways [49,50]. Similarly, we have used a variety of techniques that demonstrate extensive apoptosis after infection of MV-Edm in human HB cell lines [26]. These results are in agreement with prior work that has indicated that apoptosis is the main mechanism of death for MV-induced syncytia [51]. The exact mechanism of cell death after MV-Edm-induced syncytium formation is still unknown and should be further investigated.

One of the main advantages of MV-Edm therapy in human HB is that the tumor offers the possibility of localized treatments compared with other malignancies such as ovarian cancers, glioblastoma and prostate cancers. In most cases of HB, the tumor is located within the liver and does not have distant metastases after regular chemotherapy; also, the tumor is easily accessible using ultrasound or computed tomography (CT) guidance. HB tumors can easily be localized and injected with a therapeutic agent. In any case, the presence of anti-MV antibodies is not expected to significantly decrease efficacy. In a mouse model in which mice received passive transfer of anti-MV antibodies, Grote et al. found that intratumoral MV-Edm therapy of human lymphoma xenografts resulted in effective tumor regression without compromise through the presence of anti-MV antibodies [52]. These findings were in accordance with the results of a study of intratumoral therapy with a retrovirus in immune-competent C3H mice and of a clinical phase II study of a genetically modified adenovirus in patients with advanced head and neck cancer [53,54].

A major drawback of many cancer agents is the lack of convenient methods for monitoring the agent after administration to the patient. MV-Edm derivatives engineered to express CEA allow noninvasive tracking of the viral gene expression as well as localization of the infected tumor tissue. The advantage of this method has been well described in previous investigations [15,33]. In this study, the same result was verified in HB both in vitro and in vivo. Given the fact that Hep2G is known to intrinsically produce CEA, these cells themselves may be the source of the CEA, which would impact the serum CEA. However, the impact on the final result would be minor because all of the comparisons were carried out within the Hep2G groups instead of between the Hep2G and other groups. In addition, in the clinical practice, fewer than 10% of patients with HB can produce CEA and the amount is very little; it is likely unnecessary to worry about the confusion of intrinsic CEA.

## Conclusions

MV-CEA has potent therapeutic efficacy against human HB both in vivo and in vitro. We therefore believe that, given their antitumor activity and potential for noninvasive monitoring, engineered trackable MV derivatives warrant further investigation for their use in HB treatment. These studies have not yet been translated into a clinical trial, but should provide the foundation for future clinical developments.

## Abbreviations

(HB): Hepatoblastoma; (CEA): Carcinoembryonic antigen; (AFP): Alpha-fetoprotein; (MV-Edm): The attenuated Edmonston vaccine strain of the measles virus; (CPE): Cytopathic effect; (MOI): Multiplicity of infection.

## Competing interests

The authors declare that they have no competing interests.

## Authors’ contributions

SCZ performed the expression of CD46 and oncolytic studies and drafted the manuscript. Wei-song CAI performed the apoptosis assays and the statistical analysis. KJ performed the in vivo studies. WLW participated in the design of the study. ZWY conceived of the study, participated in its design and coordination and helped to draft the manuscript. All authors read and approved the final manuscript.

## Pre-publication history

The pre-publication history for this paper can be accessed here:

http://www.biomedcentral.com/1471-2407/12/427/prepub
